# Embryonic Medaka Model of Microglia in the Developing CNS Allowing *In Vivo* Analysis of Their Spatiotemporal Recruitment in Response to Irradiation

**DOI:** 10.1371/journal.pone.0127325

**Published:** 2015-06-10

**Authors:** Takako Yasuda, Shoji Oda, Yusuke Hibi, Satomi Satoh, Kento Nagata, Kei Hirakawa, Natsumaro Kutsuna, Hiroshi Sagara, Hiroshi Mitani

**Affiliations:** 1 Department of Integrated Biosciences, Graduate School of Frontier Sciences, Tokyo University, Bioscience Bldg., Kashiwa, Chiba, Japan; 2 Fine Morphological Analysis Group, Medical Proteomics Laboratory, Institute of Medical Science, Tokyo University, 4-6-1 Shirokanedai, Minato-ku, Tokyo, Japan; University of Cologne, GERMANY

## Abstract

Radiation therapy (RT) is pivotal in the treatment of many central nervous system (CNS) pathologies; however, exposure to RT in children is associated with a higher risk of secondary CNS tumors. Although recent research interest has focused on the reparative and therapeutic role of microglia, their recruitment following RT has not been elucidated, especially in the developing CNS. Here, we investigated the spatiotemporal dynamics of microglia during tissue repair in the irradiated embryonic medaka brain by whole-mount *in situ* hybridization using a probe for Apolipoprotein E (ApoE), a marker for activated microglia in teleosts. Three-dimensional imaging of the distribution of ApoE-expressing microglia in the irradiated embryonic brain clearly showed that ApoE-expressing microglia were abundant only in the late phase of phagocytosis during tissue repair induced by irradiation, while few microglia expressed ApoE in the initial phase of phagocytosis. This strongly suggests that ApoE has a significant function in the late phase of phagocytosis by microglia in the medaka brain. In addition, the distribution of microglia in p53-deficient embryos at the late phase of phagocytosis was almost the same as in wild-type embryos, despite the low numbers of irradiation-induced apoptotic neurons, suggesting that constant numbers of activated microglia were recruited at the late phase of phagocytosis irrespective of the extent of neuronal injury. This medaka model of microglia demonstrated specific recruitment after irradiation in the developing CNS and could provide a useful potential therapeutic strategy to counteract the detrimental effects of RT.

## Introduction

Although radiation therapy (RT) is a widely accepted treatment for many central nervous system (CNS) pathologies, there are significant detrimental effects such as an increased risk of developing secondary cancers associated with ionizing radiation. The higher incidence of secondary brain tumors among irradiated children indicates that they have greater susceptibility to radiation than do adult patients who have undergone RT. Thus, there is a need to reduce the detrimental effects of radiation for children who require RT [1, 2]. Recent research has focused on the reparative and therapeutic role of microglia, the immune cells of the brain, in various types of brain injury and disease.

Microglia in the healthy brain are highly motile cells, extending and retracting their cellular processes as they survey the microenvironment [3–5]. In fact, during the development of a normal brain, there is a vast excess of neurons compared with the ultimate adult requirement. Thus, at least half of the original cell population is normally removed by phagocytic microglia during apoptotic processes [6, 7]. Following any kind of brain damage or injury stimulus, microglia switch to an activated state, become amoeboid in morphology, and migrate toward the site of injury to engulf and eliminate neuronal debris after apoptotic cell death [8, 9]. Previous studies in the mouse have revealed a longitudinal pattern of increased numbers of microglia derived from bone marrow cells to the site of injury in the brain after exposure to radiation. By contrast, only a transient increase in microglia was seen when a restricted local area of brain was injured [10]. Previous reports using zebrafish as a model revealed the recruitment of microglia in the developing CNS when restricted local trauma was induced [11]; however, their recruitment in response to radiation exposure has not been elucidated. Here, we investigated the spatiotemporal behavior of activated microglia after irradiation in the developing brain using the medaka embryo as a model.

As vertebrate models of development, small fish have an advantage over a mouse model because of their small size and transparency, which facilitate the detection of apoptotic neurons and microglia at a whole-brain level [12, 13]. Compared with zebrafish, the medaka has more advantages for precise observation of temporal changes in the apoptotic processes after irradiation because their rate of development is slower until hatching [14]. Moreover, we took advantage of the medaka model because our previous studies revealed the spatiotemporal changes in neuronal apoptosis in the developing medaka CNS after irradiation [15–18]. We demonstrated that many apoptotic cells were induced by irradiation, especially in the marginal area of the optic tectum (OT) visualized using an acridine orange (AO) assay; however, phagocytic cells had eliminated them almost completely by the time of hatching. These irradiated embryos hatched normally and developed normally with no malformations [15, 18], suggesting that the efficient elimination of apoptotic cells by phagocytosis is essential for tissue homeostasis in these multicellular organisms, consistent with previous reports [19, 20]. Here, we investigated the spatiotemporal changes in phagocytic microglial distribution after irradiation in the developing whole brain of medaka embryos by whole-mount *in situ* hybridization (WISH) using a probe for Apolipoprotein E (ApoE).

ApoE plays important roles in lipid transport and redistribution among cells throughout the body, including the CNS. In the healthy CNS, ApoE is synthesized mainly by glial cells—in particular astrocytes—and is vital for neuronal maintenance and repair for maintaining brain homeostasis [21, 22]. ApoE has attracted considerable attention in neurobiology because emerging evidence *in vivo* suggests that one of the ApoE isoforms might induce detrimental effects, in contrast to other ApoE isoforms, which promote neural protection [23–25]. Following neuronal cell injury, upregulation of ApoE expression is induced by cholesterol, which is released from cellular debris after phagocytosis by resident macrophages in the brain [26]. A study in zebrafish using transgenic ApoE–green fluorescent protein (GFP) lines showed that the GFP-positive cells shared morphological and behavioral features with mammalian microglia, such as the capacity to phagocytose bacteria and dying neurons [11]. This suggested that an ApoE mRNA probe could be used as a marker of activated microglia in teleosts [11, 27, 28]. Our results here also showed that activated microglia expressed ApoE during phagocytosis as shown by WISH, demonstrating that ApoE-expressing cells in the medaka embryo might represent activated microglia.

Here, we demonstrated for the first time that there is a specific spatiotemporal recruitment of activated microglia during phagocytosis after irradiation in the developing normal medaka brain. Notably, the distribution of activated microglia in the whole brain changed dramatically between the initial and late phases of phagocytosis. This could not be demonstrated in a zebrafish model because of the brief period of phagocytosis. Moreover, we demonstrated the possibility that a constant level of activated microglia was recruited at the late phase of phagocytosis irrespective of the extent of neuronal injury, using medaka embryos genetically deficient in the tumor suppressor protein p53. Our findings on the recruitment of microglia after irradiation in the developing brain might provide invaluable therapeutic strategies for protecting the developing CNS of children against the detrimental effects of RT.

## Results

### Irradiation-injured neurons in the embryonic brain undergo apoptosis mainly through a p53-dependent pathway

**Fig 1:**
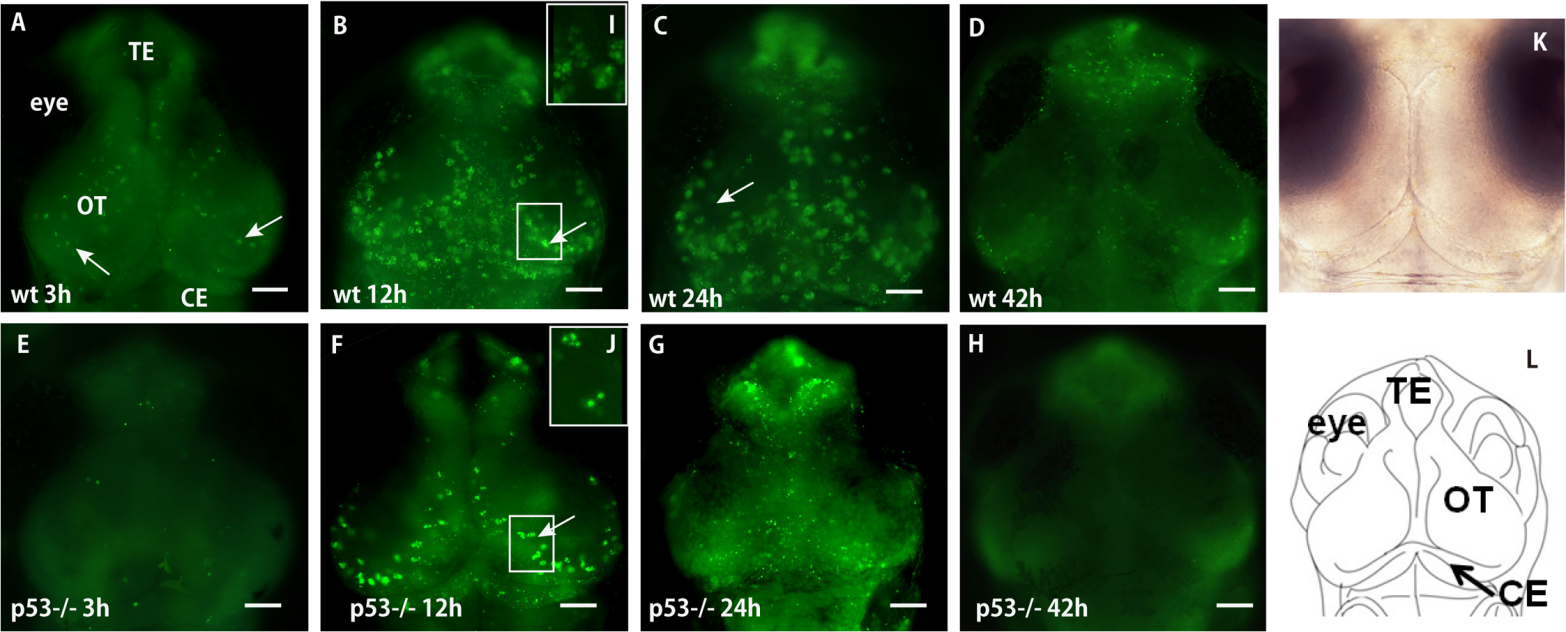
Time course of the distribution of AO-stained apoptotic cells in the irradiated brains of wt and p53^–/–^embryos. Wt (A, B, C, D) and p53^**–/–**^embryos (E, F, G, H) were irradiated with 10 Gy of gamma rays and stained with AO at 3 h (A, E), 12 h (B, F), 24 h (C, G), and 42 h (D, H) after irradiation. Images of clusters of AO-positive spots at higher magnifications (white arrows in squares in B and F) are shown in boxes (I and J) for detailed views of the rosette-shaped clusters of apoptotic neurons, which were fewer and smaller in the OT of p53^**–/–**^embryos. Also shown are a bright-field counterpart image for AO-stained fluorescence images (K) and a schematic diagram illustrating the structure of the embryonic medaka brain at stage 30 (L). CE, cerebellum; OT, optic tectum; TE, telencephalon. Scale bars = 50 μm.

**Fig 2:**
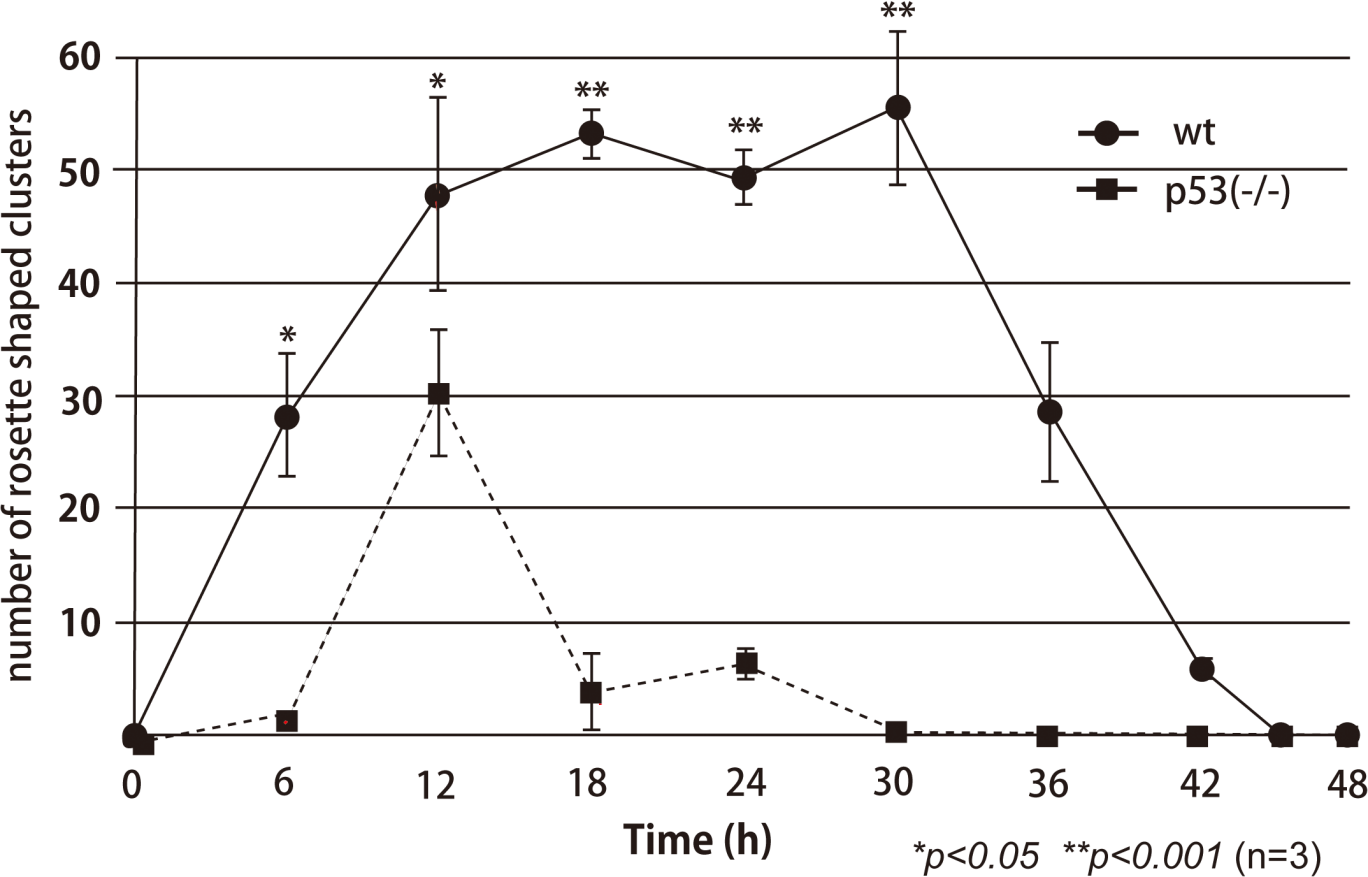
Time course of the numbers of AO-positive rosette-shaped clusters in the OT of irradiated wt and p53^–/–^embryos. The numbers of AO-positive rosette-shaped clusters in the OT were counted at various times after gamma-ray irradiation (10 Gy). Error bars show the SEM (*n* = 3). Statistical differences between the means for wt (solid line) and p53^**–/–**^embryos (broken line) were evaluated using Student’s unpaired *t* tests after *F* tests. *p < 0.05; **p < 0.01.

Irradiation by gamma rays at 10 Gy induced neural apoptosis, which was visualized as scattered AO-positive spots over the whole area of the OT in irradiated wild-type (wt) embryos at 3 h after irradiation (arrow in Fig 1A). These formed rosette-shaped clusters at 6 h after irradiation. These clusters increased in number, enlarged, and were located in the marginal area of the OT at 12 h after irradiation (Fig 1B), as reported previously [16–18]. They remained obvious in the OT up to 30 h after irradiation (Fig 1C) but had disappeared by 42 h (Figs 1D and 2). By contrast, in the irradiated brain of p53-deficient embryos, AO-positive spots were absent in the irradiated brain at 3 h after irradiation (Fig 1E), and AO-positive spots were visualized at 8 h after irradiation (data not shown). They formed fewer rosette-shaped clusters in the marginal area of the OT, at 12 h after irradiation (Fig 1F); these were clearly smaller than those observed in the brains of irradiated wt medaka (Fig 1I and 1J). Almost all of the AO-positive clusters in the OT of irradiated p53-deficient embryos disappeared within 24 h after irradiation (Figs 1G and 2), and AO-positive residual fragments of the apoptotic neurons had disappeared completely by 42 h after irradiation (Figs 1H and 2).

### Apoptotic neurons are collected for phagocytosis by microglia

**Fig 3:**
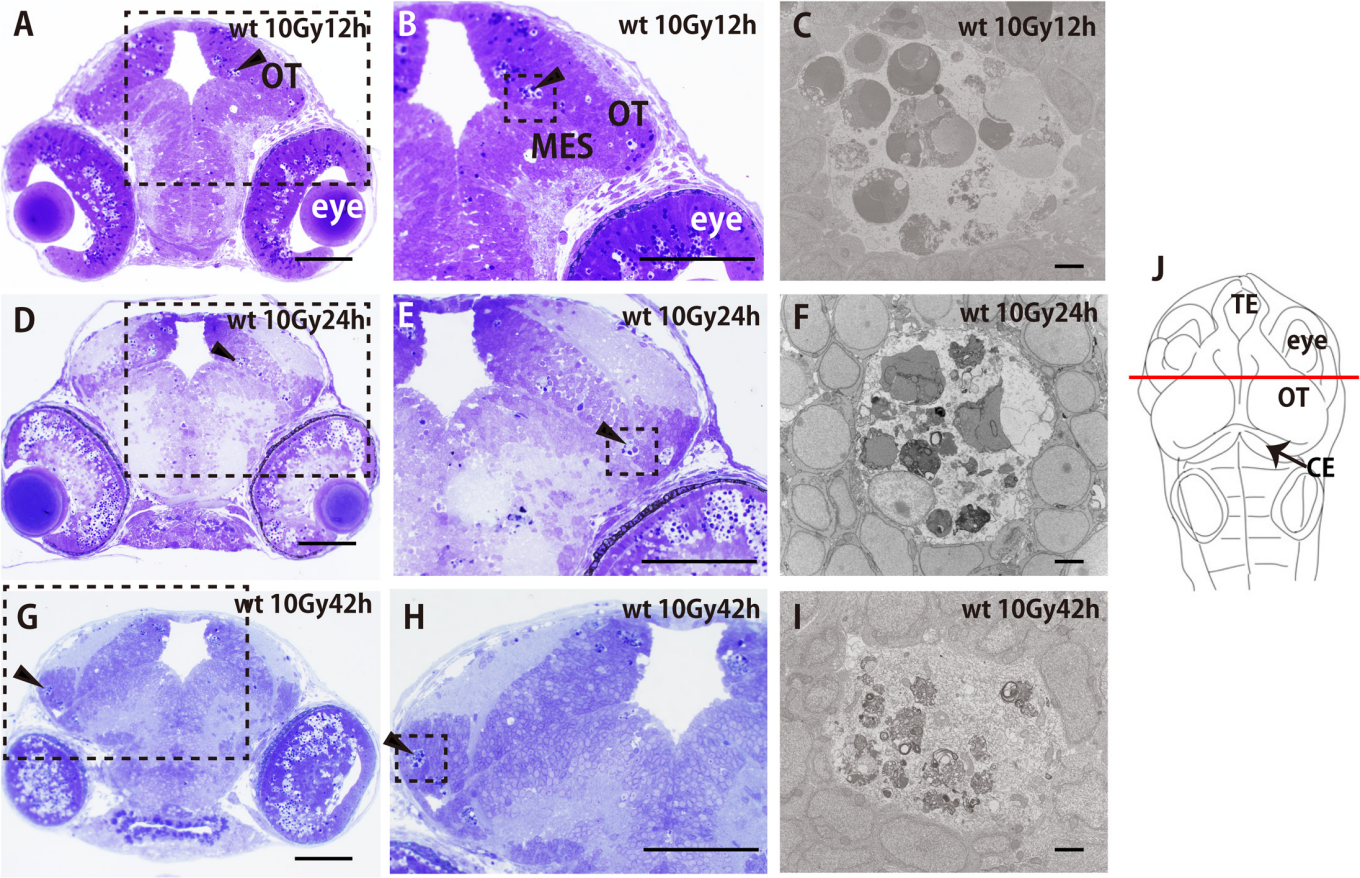
Histological analyses of the time course of neuronal damage in irradiated wt brains. Wt medaka embryos were irradiated with 10 Gy of gamma rays. Frontal sections of plastic-embedded heads were prepared in the plane that included their eyes and subjected to Nissl staining with cresyl violet. Clusters of apoptotic nuclei are shown in the irradiated wt embryos at 12 h (arrowhead in A), 24 h (arrowhead in D), and 42 h after irradiation (arrowhead in G); images at higher magnification in squares with dotted outlines in A, D, and G are shown with arrowheads in B, E, and H, respectively. Scale bars = 50 μm. Electron microscopic images of apoptotic clusters in the dotted-line boxes in B, E, and H are shown in C, F, and I, respectively. Microglia engulfed 10–15 apoptotic neurons into their phagosomes, and the nuclei of the engulfed apoptotic neurons maintained their appearance almost intact to 12 h after irradiation (C). The nuclei of the phagocytosed apoptotic neurons gradually became fragmented during the following 12 h (F). At 42 h after irradiation, degradation of apoptotic nuclei in phagosomes was almost complete (I). Frontal plastic sections (A, B, D, E, G, and H) were prepared at the level of the solid line of the embryonic brain shown in (J). CE, cerebellum; OT, optic tectum; MES, mesencephalon; TE, telencephalon. Scale bars in A, B, D, E, G, and H = 50 μm. Scale bars in C, F, and I = 2 μm.

At 12 h after irradiation, histological sections examined by light microscopy showed that single condensed nuclei of apoptotic neurons and their clusters were present in the irradiated brain of wt embryos (arrowheads in Fig 3A and 3B). Single condensed nuclei of apoptotic neurons and their clusters were also observed at 24 h after irradiation (Fig 3D), and then the numbers of condensed nuclei and their clusters decreased remarkably by 42 h (Fig 3G). Images at greater magnification showed that the clusters of condensed nuclei of apoptotic neurons were embedded in large round spaces at 12, 24, and 42 h after irradiation (arrowheads in Fig 3B, 3E and 3H). Immunological analysis on histological sections 24 h after irradiation (stage 30) using cleaved caspase-3 and an HuC/D neuronal protein antibody demonstrated that Nissl-stained condensed nuclei in large round spaces represented initial forms of apoptotic neuronal cells (S1 Fig). Electron microscopy (EM) observations showed that clustered apoptotic neurons were gathered together, and most of their nuclei appeared intact in a large round space at 12 h after irradiation (Fig 3C). The nuclei of clustered apoptotic neurons were degraded gradually, but large fragments of apoptotic nuclei debris were still present at 24 h after irradiation (Fig 3F). Subsequently, almost all of the apoptotic neurons were digested into small pieces by 42 h (Fig 3I). Because it has been accepted widely that microglia are the resident macrophages in the developing vertebrate brain [4, 11], these microscopic observations strongly suggest that the large round spaces appearing at the damaged neuronal site correspond to microglial phagosomes. EM observation of a cell projection around the clustered apoptotic cells, thought to be a part of a microglial cell (arrows in S2 Fig), also provided evidence of microglial involvement in disposing of apoptotic neurons.

**Fig 4:**
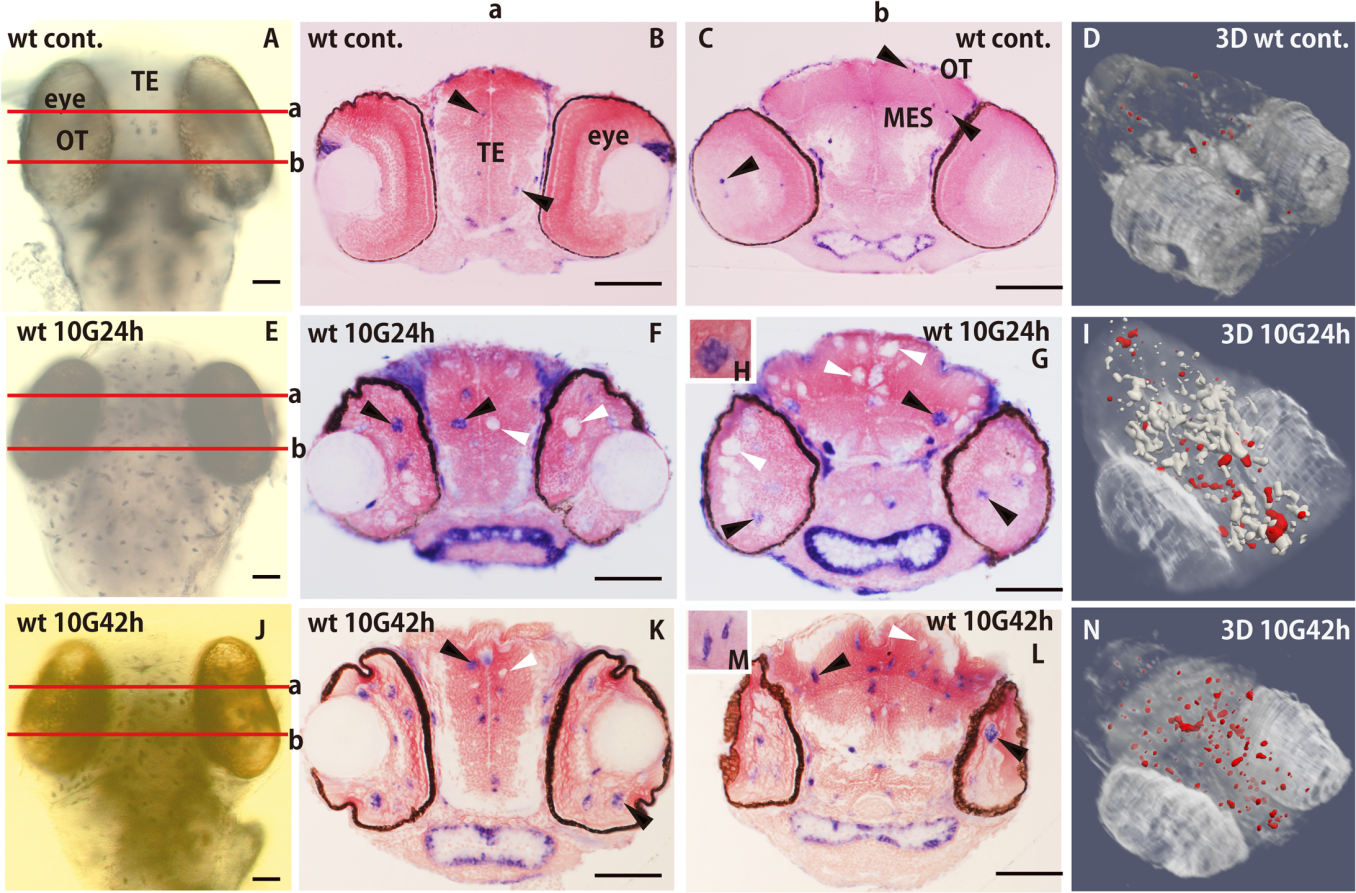
Distribution of ApoE-expressing microglia during phagocytosis shown by WISH. Activated microglia were identified as ApoE-expressing cells by WISH in nonirradiated control embryos (A), irradiated embryos at 24 h after irradiation (E), and at 42 h after irradiation (J). Frontal plastic sections including the eyes and the OT at the ‘a’ and ‘b’ levels of the brain in A, E, and J are shown in B, F, and K, and C, G, and L, respectively. A few ApoE-expressing cells were present in the retina of wt nonirradiated embryos (arrowhead in C), in the TE (arrowheads in B), and the OT (arrowhead in C). At 24 h after irradiation, hypertrophic and rounded ApoE-expressing microglia (H) appeared in the TE (arrowhead in F), retina (arrowhead in F, G), and OT (arrowhead in G). Unstained round areas were present in the TE, retina, and marginal regions of the OT (open arrowheads in F and G). At 42 h after irradiation, the number of ApoE-expressing microglia had increased markedly (arrowheads in K and L) and they showed a branched morphology (M). The numbers of unstained areas in the TE and OT (open arrowheads in K and L) decreased and they were small, not hypertrophied. Three-dimensional images were constructed from serial sections of WISH-stained nonirradiated (D), embryos at 24 h (I) and at 42 h after irradiation (N). ApoE-expressing microglia are in red and the unstained round areas appear white. MES, mesencephalon; OT, optic tectum; TE, telencephalon. Scale bars = 50 μm.

To clarify the critical roles of microglia in the clearance of damaged neurons in the developing brain, the expression of ApoE mRNA, as a putative marker for activated microglia [11, 28], was examined by WISH. Upregulation of ApoE expression was observed at 24 and 42 h after irradiation (Fig 4E and 4J); by contrast, no ApoE expression outside the retina was observed 12 h after irradiation (S3 Fig). In the brains of nonirradiated wt embryos, only a few ApoE-expressing microglia were present in the retina, telencephalon (TE), and OT (closed arrowheads in Fig 4B and 4C) without irradiation (Fig 4A and 4D; S1 Movie).

At 24 h after irradiation, hypertrophied and rounded ApoE-expressing microglia (Fig 4H) appeared in the retina (closed arrowheads in Fig 4F and 4G) and in the OT (closed arrowhead in Fig 4G), while many round ApoE-unstained areas, which were also supposed to be activated microglia, appeared in the TE and OT (open arrowheads in Fig 4F and 4G).

Three-dimensional (3D) images of ApoE-expressing microglia (red dots in Fig 4I) and ApoE-unstained microglia (white regions in Fig 4I) constructed from serial sections of WISH-stained irradiated embryos (S2 Movie) demonstrated that only a small proportion of activated microglia could express ApoE (S2 Movie, Fig 4I). Moreover, the distributions that combined ApoE-unstained (green area in S4C Fig) and ApoE-expressing (red area in S4C Fig) microglia were identical to those of AO-positive apoptotic neurons in the OT (S4A Fig). These 3D images of ApoE distribution demonstrated two types of microglial subpopulations—ApoE-expressing and ApoE-unstained microglia—that were engaged in phagocytotic activity at the initial phase of phagocytosis, as confirmed by histological examination of EM (Fig 3F).

To clarify whether ApoE-unstained cells might be microglia activated for phagocytosing apoptotic neurons, we performed WISH 24 h after irradiation with leucocyte-specific plastin (L-plastin) mRNA as a marker for early macrophages, which are commonly accepted as the source of microglia [28–30]. L-plastin was distributed marginal area in the OT, which was identical to that of AO-positive apoptotic neurons 24 h after irradiation (S5C and S5D Fig). This result strongly suggests that activated microglia for phagocytosing apoptotic neurons would also be present at ApoE-unstained areas.

Histology of serial sections of WISH-stained embryos 42 h after irradiation and 3D images constructed from them demonstrated that a remarkably increased number of ApoE-expressing microglia had spread through the whole brain at this time (filled arrowheads in Fig 4K and 4L, red dots in Fig 4N, and S3 Movie). Their morphology changed to being small and highly branched (Fig 4M). By contrast, the numbers of ApoE-unstained areas in the TE and OT (open arrowheads in Fig 4K and 4L) obviously decreased and they were smaller than those at 24 h after irradiation. This result suggests that apoptotic neuronal cells were almost completely removed by microglial phagocytosis at 42 h after irradiation, corresponding to the last phase of phagocytosis during tissue repair confirmed by EM observations (Fig 3I). These results demonstrated that ApoE-expressing microglia had spread through the whole brain during the last phase of phagocytosis, regardless of the damaged neuronal site (Fig 4N; S3 Movie).

### The distribution of ApoE-expressing microglia varies dynamically depending on progressive neuronal degradation in the microglial phagosomes

**Fig 5:**
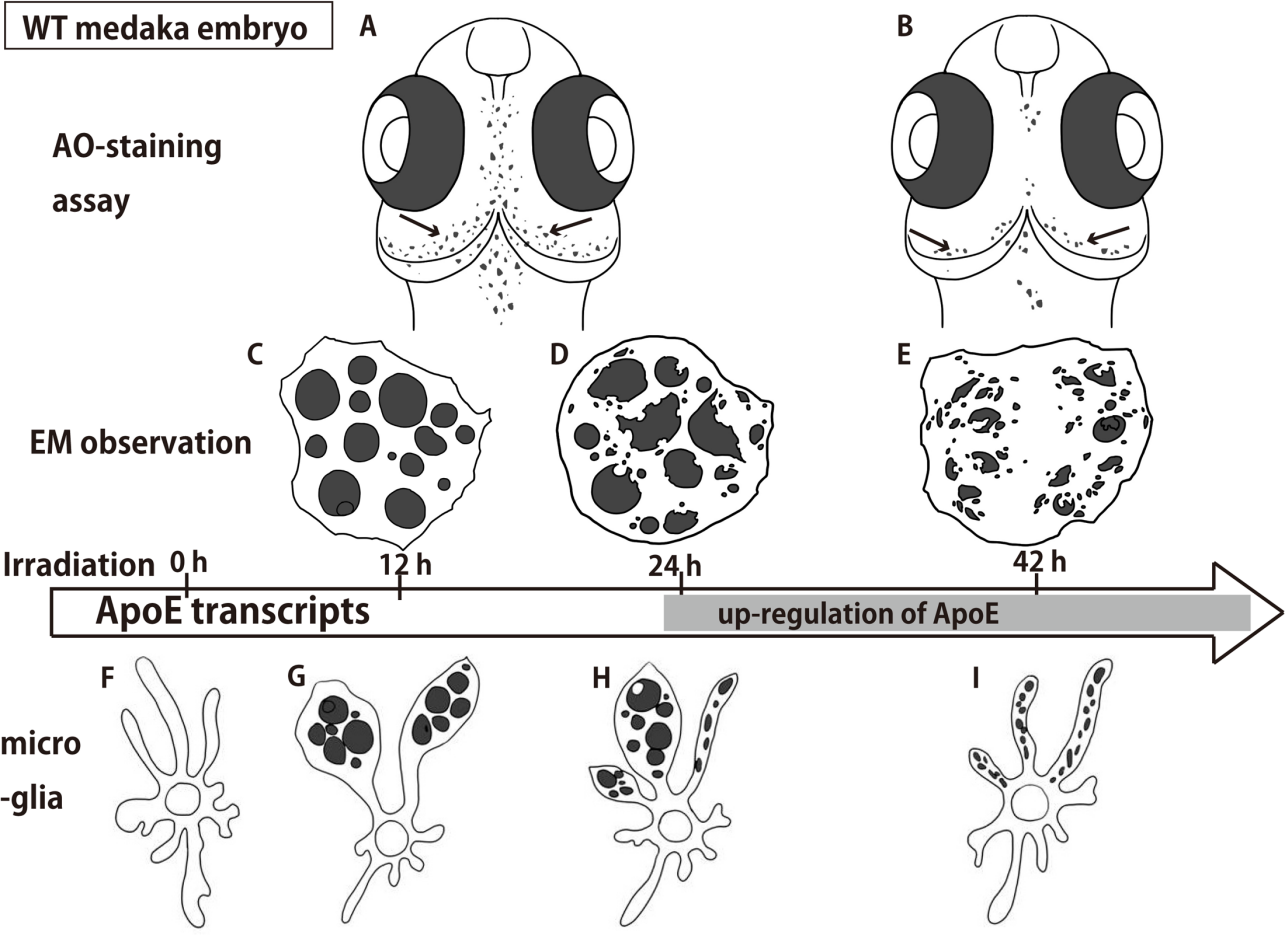
Summary of changes in the radiation-induced activated microglia in the irradiated wt medaka brain. Microglia (F) engulfed apoptotic neurons into their phagosomes within 12 h after irradiation (G) and underwent hypertrophy at a marginal region of the OT, appearing as rosette-shaped clusters by AO staining (A) and as a cluster of intact apoptotic nuclei in a microglial phagosome by EM (C). At 24 h after irradiation, microglia had partially digested the apoptotic neurons (D, H) and began to express ApoE. At 42 h after irradiation, microglia had completely digested the apoptotic neurons in their phagosomes (E, I) and apoptotic neurons were no longer stained by AO (B), whereas microglia continued to express ApoE. OT, optic tectum.

Three-dimensional imaging of the distribution of ApoE-expressing microglia in the irradiated embryonic brain clearly demonstrated that ApoE-expressing microglia were abundant only in the late phase of phagocytosis during tissue repair induced by irradiation, while few microglia expressed ApoE in the initial phase of phagocytosis (Fig 4). The precise stage was confirmed by the presence of degraded apoptotic neurons in microglial phagosomes observed by EM (Fig 3). At 12 h after irradiation when the AO-positive rosette-shaped clusters were obvious (Figs 1B and 5A), most of the nuclei of apoptotic neurons appeared intact in the microglial phagosomes (Figs 3C and 5C), when upregulation of ApoE expression was not observed (S3 Fig). Then, at 24 h after irradiation when the AO-positive rosette-shaped clusters were still obvious (Figs 1C and 5A), the nuclei of phagocytosed apoptotic neurons were degraded but large debris fragments from the nuclei were still present in microglial phagosomes (Figs 3F and 5D), when only a small proportion of activated microglia expressed ApoE (Fig 4F–4I; S2 Movie). Subsequently, at 42 h after irradiation when AO failed to stain the clusters of apoptotic neurons (Figs 1D and 5B), almost all of the apoptotic neurons were digested into small pieces in the microglial phagosomes (Figs 3I and 5E), and increased numbers of ApoE-expressing microglia had spread through the whole brain (Fig 4K–4N; S3 Movie). These findings led us to the hypothesis that ApoE has a significant function in the late phase of phagocytosis by microglia in the medaka brain, shown as schematic images in Fig 5.

#### ApoE-expressing microglia spread throughout the whole brain irrespective of the considerably reduced number of apoptotic neurons in irradiation-injured p53-deficient embryos

**Fig 6:**
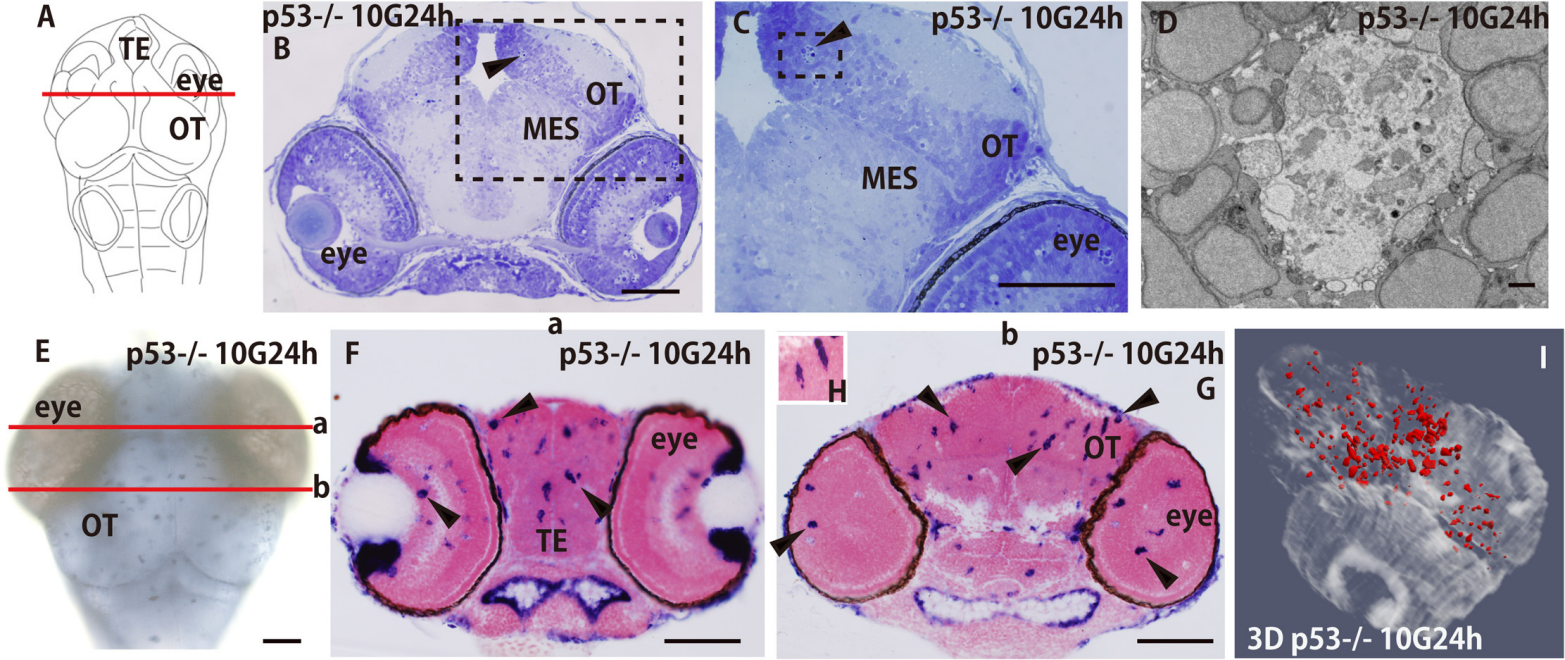
Increased numbers of ApoE-expressing cells were present in p53^-/-^ embryos at the late phase of phagocytosis. The p53^**–/–**^embryos were irradiated with 10 Gy of gamma rays and frontal sections of heads including the eyes embedded in plastic resin were cut at the level of the solid line in A and subjected to Nissl staining with cresyl violet. Clusters of apoptotic nuclei were present at 24 h after irradiation (arrowhead in B) and images at higher magnification of the squares with dotted outlines in B are shown in C with an arrowhead. An EM image of phagocytosed apoptotic nuclei in a microglial phagosome (D) showed complete digestion of apoptotic nuclei in microglial phagosome as in wt embryos at 42 h after irradiation (Fig 3I). Activated microglia were identified as ApoE-expressing cells by WISH in irradiated p53^**–/—**^embryos 24 h after irradiation (E). Frontal plastic sections including the eyes and the OT of WISH-stained p53^**–/–**^embryos (stage 30) at the ‘a’ and ‘b’ levels of the brain in E are shown in F and G, respectively. The numbers of ApoE-expressing microglia increased (arrowheads in F and G) and they showed a branched morphology, not hypertrophy (a higher magnification is shown in H). The 3D reconstructed images showed dramatically increased numbers of ApoE-expressing cells in the irradiated p53^**–/–**^embryos (I), as in wt embryos 42 h after irradiation (see Fig 4N). MES, mesencephalon; OT, optic tectum; TE, telencephalon. Scale bar in (D) = 2 μm; Scale bars in (B–G) = 50 μm.

Clusters of apoptotic nuclei were also present in the brain of p53-deficient embryos 24 h after irradiation (arrowhead in Fig 6B), although they were much fewer than in the brains of wt embryos. Presumably, this was because there was much less induction of apoptosis as shown by the AO assays (Figs 1 and 2). Highly magnified images show that the clusters of condensed nuclei of apoptotic neurons were embedded in the large round spaces in the brains of p53-deficient embryos (arrowhead in Fig 6C), as also seen in the brains of wt embryos (Fig 3B, 3E and 3H).

EM observations on irradiated p53-deficient brains at 24 h after irradiation showed that almost all of the apoptotic neurons were digested into small pieces in the microglial phagosomes (Fig 6D) as in the wild-type brain 42 h after irradiation (Fig 3I). This result indicated that the irradiated p53-deficient brains at 24 h after irradiation also corresponded to the late phase of phagocytosis as in wt brains at 42 h after irradiation.

In nonirradiated p53-deficient embryos, a few ApoE-expressing microglia were present in the retina, TE, and OT (arrowheads in S6B, S6C Fig) as in wt embryos (Fig 4A–4D). At 24 h after irradiation, the numbers of ApoE-expressing microglia increased dramatically (Fig 6E and arrowheads in Fig 6F and 6G). The microglia were not hypertrophied at this time; instead, they were already small and showed a branched morphology (a high magnification image is shown in Fig 6H) similar to those of wt embryos 42 h after irradiation (Fig 4M). The 3D images of irradiated p53-deficient brains at 24 h after irradiation (Fig 6I, S4 Movie) clearly show that ApoE-expressing microglia had spread through the whole brain as seen in wild-type embryos at 42 h after irradiation (Fig 4N, S2 Movie) at the late phase of phagocytosis, irrespective of the considerably fewer induced apoptotic neurons in the irradiation-injured p53-deficient brains.

## Discussion

There is a need to diminish the risk of secondary brain tumors among children who have undergone RT [1, 2]. However, the recruitment of activated microglia in response to irradiation has not been elucidated, especially in the developing CNS. In addition, it is essential that apoptotic neurons are recognized quickly and phagocytosed by microglia, thus preventing the diffusion of apoptotic degradation products into surrounding tissues and disrupting brain homeostasis [19, 20]. When neuronal disposal by microglia is uncontrolled, microglial-mediated neuroinflammation has been implicated in many neuronal disorders and can cause neurodegenerative disorders such as Alzheimer’s, Parkinson’s, and Huntington’s diseases [29, 31]. Here, we demonstrated for the first time the spatiotemporal involvement of activated microglia after irradiation in the developing CNS manifested as ApoE-expressing cells in the whole brain using medaka embryos. Previous studies using zebrafish embryos revealed the recruitment of these cells in the developing CNS only when a restricted local area of the brain was injured [11]. Our results demonstrated that the recruitment of activated microglia following irradiation continued in the long term even when injured neurons had been largely eliminated by apoptosis at the late phase of phagocytosis (Fig 5). Moreover, they were distributed through the whole brain, not only at the damaged site. Previous *in vivo* studies using a mouse model have reported similar results; namely, that a long-term increase in microglial involvement was induced in response to irradiation, in contrast to a transient increase when local injury was induced in the brain [10]. These results were consistent with our hypothesis that the involvement of activated microglia in response to irradiation is controlled in a common manner between the mature and embryonic brain.

Our results using p53-deficient embryos showed that ApoE-expressing microglia became scattered through the whole brain at the late phase of phagocytosis—as in wt irradiated embryos—irrespective of the relatively low number of radiation-induced apoptotic neurons (Figs 1, 2 and 6). This suggests that constant numbers of activated microglia must be recruited at the late phase of phagocytosis, independent of the extent of neuronal injury in the brain. This finding would not be in accord with a result reported for the mouse brain, which demonstrated a dose-dependent increase of activated microglia after irradiation [10]. To confirm this finding in p53-deficient medaka embryos, we need further investigations on whether activated microglia are recruited at a constant level at the late phase of phagocytosis following different doses of radiation.

Here, the distribution of activated microglia was examined with an ApoE mRNA probe, which was previously confirmed by WISH in a zebrafish model as a marker of activated microglia [11, 28, 30]. However, our 3D images of ApoE distributions demonstrated that there are two types of microglial subpopulations—ApoE-expressing and ApoE-unstained microglia—engaged in phagocytosis. (Fig 4F–4I, S2 Movie, S4 Fig). Moreover, to clarify whether ApoE-unstained cells might be microglia activated for phagocytosing apoptotic neurons, we performed WISH with L-plastin mRNA. This showed clearly that the distribution of L-plastin was identical to that of AO-stained apoptotic neurons (S5 Fig), strongly suggesting that ApoE-unstained cells are indeed activated microglia.

To evaluate the distribution of ApoE-expressing microglia, 3D images were more suitable than quantitative assessment on histological sections because the hypertrophied microglia extended over 2–3 successive sections (8 μm) when involved in engulfing many apoptotic neurons in their phagosomes. Our 3D analysis of ApoE-expressing microglia showed that ApoE had a significant function in the late phase of phagocytosis by microglia, probably because ApoE was only required when microglia had almost finished digesting apoptotic neuronal cells for the clearance of lipoprotein and cholesterol released from apoptotic cellular debris, as reported previously [26]. Thus, the slower morphogenesis of the medaka brain compared with the rapid embryogenesis of zebrafish allowed us to observe precise temporal changes in microglial involvement between the initial and late phases of phagocytosis, which would be difficult to confirm in a zebrafish model because of their brief period of phagocytosis [14]. In addition, we demonstrated that activated microglia were present at the injured neuronal site during the initial phase of phagocytosis; however, they became scattered through the whole brain at the late phase, independently of the injured neuronal site. Previous reports on the normal mouse brain showed that microglia are highly active even when they are resting; they continually survey their microenvironment with extremely motile processes and protrusions, suggesting that they are involved in surveying the whole brain [3, 4]. Once local brain injury is induced, microglial branched processes change their configuration to a rounded amoeboid morphology and migrate to the injured site to remove damaged cells and their debris [8, 29, 32]. Here, many highly branched microglia were present even after phagocytosis had finished, suggesting that the activated microglia continue to undergo vigilant monitoring in the long term to protect brain homeostasis even after the removal of apoptotic cells.

We wish to stress the usefulness of the medaka model for monitoring microglia, compared with mouse models. One of the advantages of our model is that we could make direct observations of activated microglia *in vivo* throughout the whole brain because of its small size and transparency. In addition, we could construct 3D images of microglial distribution in whole brain relatively easily because we needed to prepare fewer than 50 serial sections; this would be much more laborious for the mouse brain.

Here, using 3D images of medaka embryos, we have demonstrated for the first time that the distribution of ApoE-expressing microglia changed dramatically at the initial and late phases of phagocytosis in the developing brain after irradiation. Our findings of microglial involvement in response to irradiation in the developing CNS might provide a model of apoptotic neuronal degradation and enable strategies for treating the CNS in children to counteract the detrimental effects of RT.

## Materials and Methods

### Ethics

This research was conducted using protocols approved by the Animal Care and Use Committee of the University of Tokyo (permit number: C-09-01). All surgery on embryos was performed using chilling as anesthesia, and all efforts were made to minimize suffering.

### Fish and embryos

An Hd-rR inbred strain of medaka (*Oryzias latipes*), established from a southern population [33], was kept in our laboratory. The p53-deficient fish were generated originally by inducing targeted local lesions in fish genomes with a genetic background based on the CAB Direct database [34; http://www.cabdirect.org] and backcrossed four times with Hd-rR fish to establish a p53-deficient strain on an Hd-rR genomic background [35]. The fish were kept at 26–28°C under a 14 h light and 10 h dark cycle, and fed on a powdered diet (TetraMin, Tetra Werke, Melle, Germany) and brine shrimp (*Artemia franciscana*) three times per day.

Female medaka spawn eggs every morning. Egg clusters were collected and rubbed between two small pieces of paper towel to remove filaments on the chorion; the isolated eggs were then incubated in a petri dish filled with 7 ml of distilled water containing 10^–5^% (w/v) methylene blue at 26–29°C. The developmental stages of the embryos are described according to Iwamatsu [36].

### Irradiation

Embryos at stage 28 (30-somite stage, 64 h after fertilization) were irradiated with gamma rays emitted by ^137^Cs (10 Gy, Gammacell 3000Elan, MDS Nordion, Ottawa, Canada) at a dose rate of 10 Gy/min at room temperature in a plastic tub containing water. Medaka embryos at stage 28 correspond approximately to early fetal stage human embryos (approximately 8–15 weeks postovulation) [37].

### Quantification of apoptosis by AO staining assay

Acridine orange (Sigma-Aldrich, St Louis, MO, USA), a single-strand DNA intercalating vital dye, selectively stains the nuclei of apoptotic cells and does not significantly label those of necrotic cells [38, 39]. To quantify radiation-induced apoptosis in the developing OT at various times after irradiation, the irradiated embryos were stained with AO (17 μg/ml) as described [16, 18]. The AO-stained embryos were observed using a fluorescence microscope (BX50, Olympus, Tokyo, Japan) with an appropriate filter (U-MGFPHQ, Olympus), 460–480 nm excitation, and 495–540 nm emission wavelengths (excited in blue and fluorescence emission in green), equipped with a digital camera (DP70, Olympus). AO-stained rosette-shaped clusters were counted at a focal plane where the maximum number of clusters was seen by fluorescence microscopy. Statistical analysis comparing mean data was conducted using Student’s unpaired *t* tests following *F* tests; *p* < 0.05 was considered to be statistically significant.

### Histology and Electronmicroscope (EM)

Medaka embryos were anesthetized by chilling and fixed in 4% (w/v) paraformaldehyde in 0.1 M phosphate buffer overnight at 0–4°C. The fixed embryos were dehydrated in an ethanol series, embedded in plastic resin (Technovit 8100, Heraeus Kulzer, Wehrheim, Germany), and sectioned frontally into a complete series of serial sections (8 μm thick), as described [15, 17]. The sections were Nissl-stained with cresyl violet for light microscopy. For EM observations, the embryos were fixed and embedded in plastic resin as described [16, 35]. Ultrathin sections were cut, stained with uranyl acetate and lead citrate, and examined with a Hitachi H-7500 electron microscope operated at 80 kV (Hitachi Ltd., Tokyo, Japan).

### Immunohistochemistry

The embryo and adult brains used for immunohistochemistry were prepared as reported [17]. Serial sections (30 μm thick) cut on a cryostat were prepared, blocked by incubation in phosphate-buffered saline (PBS) containing normal goat serum for 30 min at room temperature, washed in PBS and incubated with a polyclonal anti-cleaved caspase-3 antibody (9661S, Cell Signaling Technology, Danvers, MA, USA) (1:200) and with a monoclonal anti-HuC/D antibody (A21271, Molecular Probes, Eugene, OR, USA) for 3 h at room temperature. The sections were further incubated with secondary antibodies conjugated with Alexa-488 (A11001, Invitrogen, Carlsbad, CA, USA) and Alexa-546 (A11035, Invitrogen) (1:1000) for fluorescent images and counterstained with DAPI. Fluorescent images were obtained using a confocal microscope (FluoView FV1000, Olympus).

### Whole-mount in situ hybridization (WISH)

The sequences of the medaka ApoE and L-plastin genes were obtained from the Ensembl Genome Browser database (http://asia.ensembl.org/index.html). A DNA fragment of these genes was amplified using polymerase chain reaction (PCR) from Hd-rR cDNA, with primer pairs as follows: ApoE forward, 5’–CGAAACCATGACTGAGGTGA–3’ and reverse, 5’–AACCCTCAAAAACCCCAAGT–3’; L-plastin forward, 5’–ACCTTCAGGAAAGCCATCAA–3’ and reverse, 5’–ATTCACACCTAGAGAGTTCATCCA–3’. The PCR reactions were performed for 30 cycles at 95°C for 30 s, 60°C (ApoE) and 55°C (L-plastin) for 30 s, and 72°C for 1 min. The amplified ApoE and L-plastin cDNA sequences were cloned into pCR4-TOPO, digested with NotI, and transcribed *in vitro* with T3 polymerase to prepare DIG-labeled RNA probes for WISH. Embryos stained with the ApoE probe at 0, 12, 24, and 42 h after irradiation were embedded in Technovit 8100, serially sectioned (8 μm), and counterstained with 0.5% neutral red (Muto Pure Chemicals, Tokyo, Japan) before microscopy.

### Reconstruction and computer visualization of the 3D expression pattern of ApoE

Serial histological images of WISH-processed embryos were acquired with a microscope (BX50, Olympus) equipped with a 20 × objective lens and a digital camera (DP70, Olympus). To align the sections, each image was rotated and shifted using Photoshop (Adobe Systems, Mountain View, CA, USA) and the StackReg plug-in [40] for ImageJ (http://imagej.nih.gov/ij/). Following gray-scale conversion, the ApoE-expressing regions were segmented manually as colored spots with Photoshop using a pen tablet. Serial gray-scale images and segmented images were stacked as X–Y–Z images on ImageJ and exported as a Visualization Tool Kit (VTK) file using a KbiVtk plug-in (http://hasezawa.ib.k.u-tokyo.ac.jp/zp/Kbi/ImageJKbiPlugins). Once the VTK files had been exported, the 3D expression pattern of ApoE was displayed using a volume rendering method with the visualization software ParaView, version 3.14 (http://www.paraview.org/). We captured the rendering window of ParaView and encoded it into movie files (S1–S4 Movies).

### Data availability

All data underlying the findings described in this manuscript are fully available without any restriction.

## Supporting Information

S1 Fig Apoptotic cells in the OT 24 h after irradiation were revealed by immunohistochemistry. Apoptotic cells in the OT of the irradiated embryonic brain 24 h after irradiation (stage 30) were revealed by immunohistochemistry using an anti-cleaved caspase-3 antibody. The frontal sections including the OT and eyes were counterstained with an anti-HuC/D antibody (green in A–F) and DAPI (G–I). No positive cells were present in nonirradiated embryonic brains at stage 30 (A). Higher magnified images of the squares with dotted outlines in A and B are shown in B and C, respectively. Clusters of apoptotic nuclei in the round holes at 24 h after irradiation are shown with arrowheads in D and G. Higher magnified images of clustered apoptotic cells in squares with dotted outlines in D and G are shown with arrowheads in E and H; those in squares with dotted outlines in E and H are shown with arrowheads in F and I, respectively. OT, optic tectum; MES, mesencephalon. Scale bars = 50 μm.
(TIF)

S2 Fig Microglial projection by electron microscopy. A microglial projection (arrow) was observed around the clustered apoptotic neurons (arrowheads) in irradiated wild-type embryos 12 h after irradiation. Scale bar = 2 μm.
(TIF)

S3 Fig Apolipoprotein E (ApoE) expression by whole-body *in situ* hybridization (WISH) in wild-type embryos 12 h after irradiation. Apolipoprotein E (ApoE) expression identified by WISH in wild-type embryos 12 h after irradiation was observed only in the retina (arrows). Scale bars = 50 μm.
(TIF)

S4 Fig 3D images of ApoE-unstained microglia and ApoE-expressing microglial distributions in irradiated wild-type embryos 24 h after irradiation. 3D reconstructed images of ApoE-unstained microglia exhibited round holes in sections (green area in C), and ApoE-expressing regions in irradiated wild-type embryos 24 h after irradiation (red dots in C). The ApoE-unstained (green area in C) and ApoE-expressing (red dots in C) microglial distributions together were identical to the area of AO-positive apoptotic neurons (arrows in A) in the OT region outlined in blue in A and B. AO; acridine orange; OT, optic tectum. Scale bars = 50 μm.
(TIF)

S5 Fig Distribution of L-plastin-expressing microglia by WISH. A schematic diagram illustrating the structure of the embryonic medaka brain at stage 30 (A). Activated microglia were examined for the expression of L-plastin by WISH in nonirradiated control embryos (B) and in irradiated embryos 24 h after irradiation (D). L-plastin was localized in a marginal area of the OT (arrows in D), identical to that of AO-positive apoptotic neurons 24 h after irradiation (arrows in C). AO; acridine orange; OT, optic tectum. Scale bars = 50 μm.
(TIF)

S6 Fig ApoE-expressing cells by WISH in nonirradiated p53^-/-^ embryos. ApoE-expressing cells were demonstrated by WISH in nonirradiated p53^-/-^ embryos. Frontal plastic sections including the eyes and the OT of WISH-stained p53^–/–^embryos at the ‘a’ and ‘b’ levels of the brain are shown in B and C, respectively. A small number of ApoE-positive cells were present in the retina (arrowheads in A and B), telencephalon (TE; arrowheads in A), and optic tectum (OT; arrowhead in B). Scale bars = 50 μm.
(TIF)

S1 Movie 3D images of ApoE in a nonirradiated wt embryo. This video shows ApoE expression (red dots) in the nonirradiated wt brain, excluding the eyes.
(MOV)

S2 Movie 3D images of ApoE and ApoE-unstained vacuole on histological sections in an irradiated wt embryo 24 h after irradiation. This video shows 3D images of ApoE (red dots) in an irradiated wt embryo 24 h after irradiation (excluding the eyes) and a ApoE-unstained large vacuole representing microglia undergoing phagocytosis of apoptotic neurons (white area).
(MOV)

S3 Movie 3D images of ApoE in an irradiated wt embryo 42 h after irradiation. This video shows 3D images of ApoE in the brain (excluding the eyes) of an irradiated wt embryo 42 h after irradiation (red dots).
(MOV)

S4 Movie 3D images of ApoE in an irradiated p53^-/-^ embryo 24 h after irradiation. This video shows 3D images of ApoE in the brain (excluding the eyes) of an irradiated p53^-/-^ embryo 24 h after irradiation (red dots).
(MOV)
