# Supplementary figures and images for: Embryonic Medaka Model of Microglia in the Developing CNS Allowing In Vivo Analysis of Their Spatiotemporal Recruitment in Response to Irradiation
Source: PLoS One. 2015 Jun 10;10(6):e0127325. doi: 10.1371/journal.pone.0127325 (PMC4465025; doi:10.1371/journal.pone.0127325)

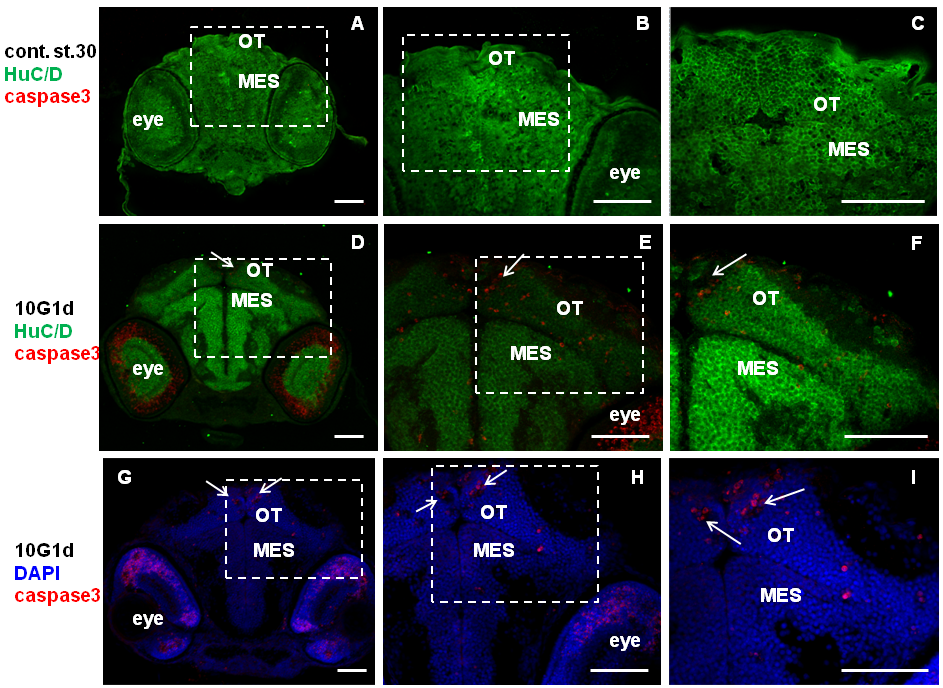

Supplement: S1 Fig — Apoptotic cells in the OT of the irradiated embryonic brain 24 h after irradiation (stage 30) were revealed by immunohistochemistry using an anti-cleaved caspase-3 antibody. The frontal sections including the OT and eyes were counterstained with an anti-HuC/D antibody (green in A–F) and DAPI (G–I). No positive cells were present in nonirradiated embryonic brains at stage 30 (A). Higher magnified images of the squares with dotted outlines in A and B are shown in B and C, respectively. Clusters of apoptotic nuclei in the round holes at 24 h after irradiation are shown with arrowheads in D and G. Higher magnified images of clustered apoptotic cells in squares with dotted outlines in D and G are shown with arrowheads in E and H; those in squares with dotted outlines in E and H are shown with arrowheads in F and I, respectively. OT, optic tectum; MES, mesencephalon. Scale bars = 50 μm. (TIF) [file pone.0127325.s001.tif]

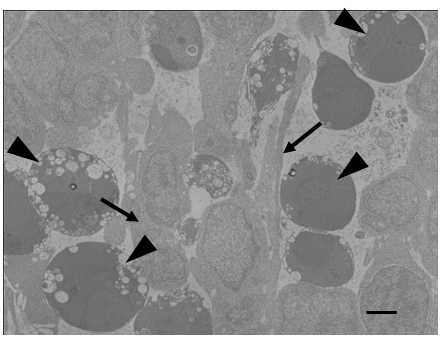

Supplement: S2 Fig — A microglial projection (arrow) was observed around the clustered apoptotic neurons (arrowheads) in irradiated wild-type embryos 12 h after irradiation. Scale bar = 2 μm. (TIF) [file pone.0127325.s002.tif]

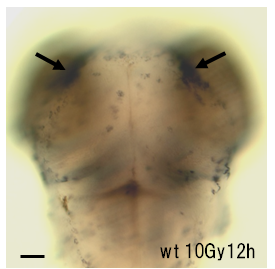

Supplement: S3 Fig — Apolipoprotein E (ApoE) expression identified by WISH in wild-type embryos 12 h after irradiation was observed only in the retina (arrows). Scale bars = 50 μm. (TIF) [file pone.0127325.s003.tif]

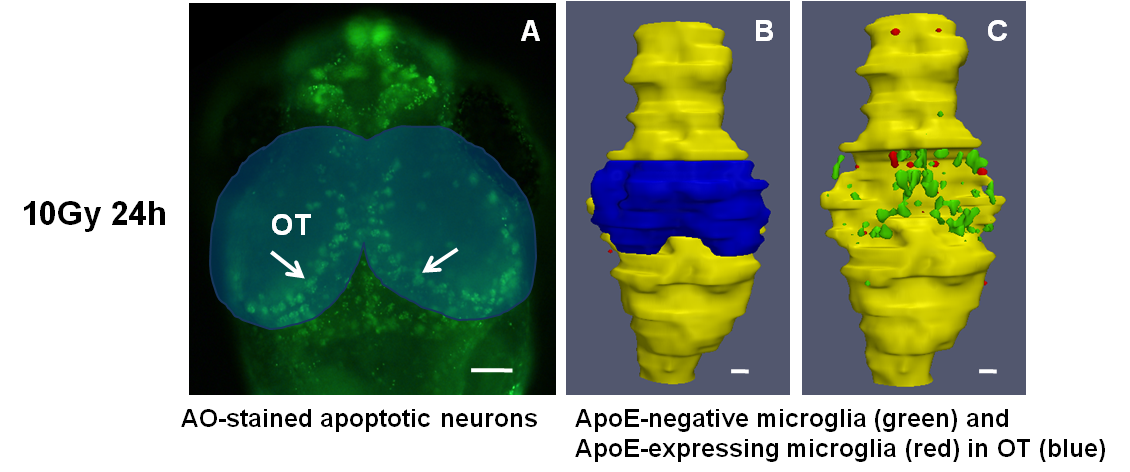

Supplement: S4 Fig — 3D reconstructed images of ApoE-unstained microglia exhibited round holes in sections (green area in C), and ApoE-expressing regions in irradiated wild-type embryos 24 h after irradiation (red dots in C). The ApoE-unstained (green area in C) and ApoE-expressing (red dots in C) microglial distributions together were identical to the area of AO-positive apoptotic neurons (arrows in A) in the OT region outlined in blue in A and B. AO; acridine orange; OT, optic tectum. Scale bars = 50 μm. (TIF) [file pone.0127325.s004.tif]

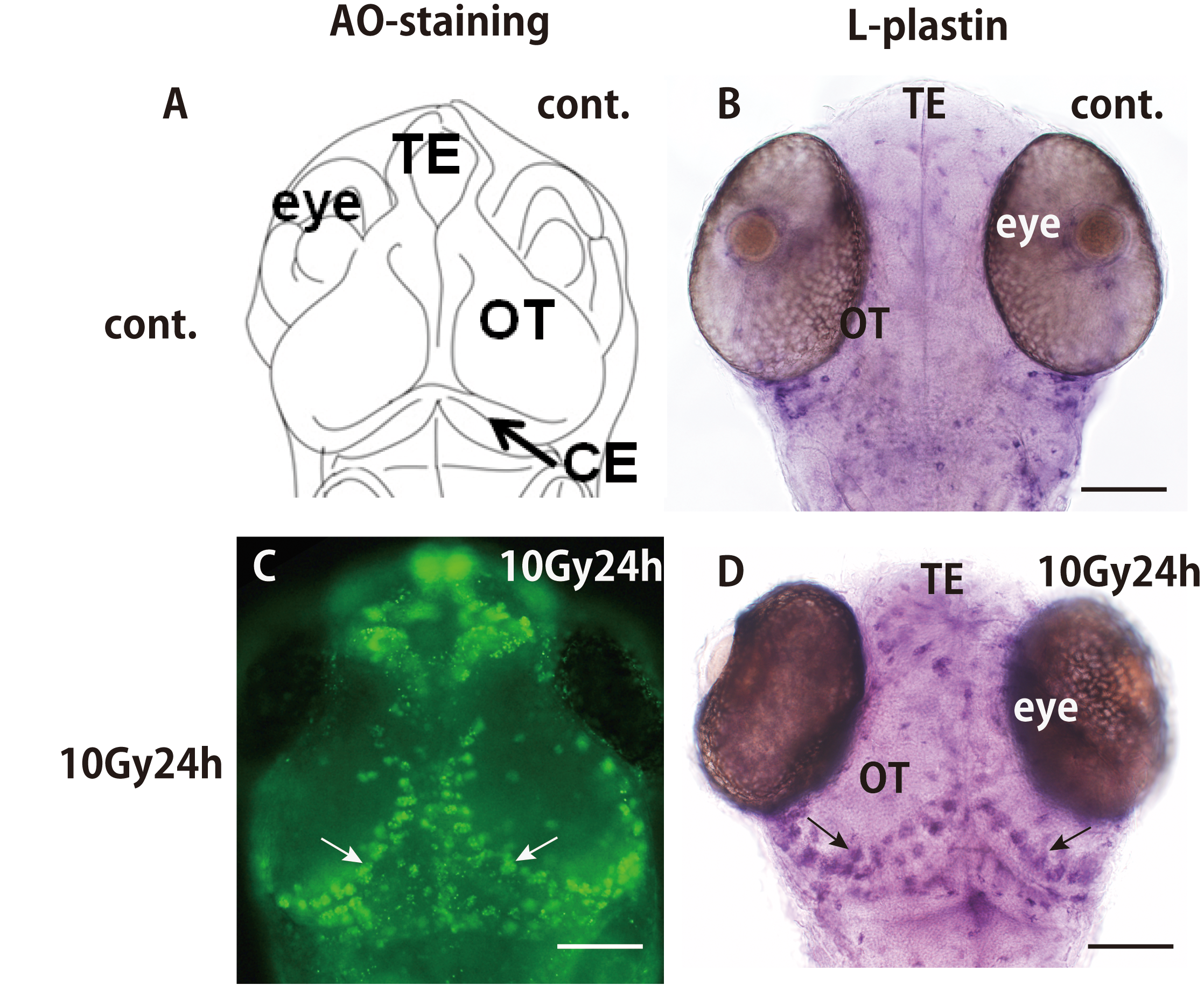

Supplement: S5 Fig — A schematic diagram illustrating the structure of the embryonic medaka brain at stage 30 (A). Activated microglia were examined for the expression of L-plastin by WISH in nonirradiated control embryos (B) and in irradiated embryos 24 h after irradiation (D). L-plastin was localized in a marginal area of the OT (arrows in D), identical to that of AO-positive apoptotic neurons 24 h after irradiation (arrows in C). AO; acridine orange; OT, optic tectum. Scale bars = 50 μm. (TIF) [file pone.0127325.s005.tif]

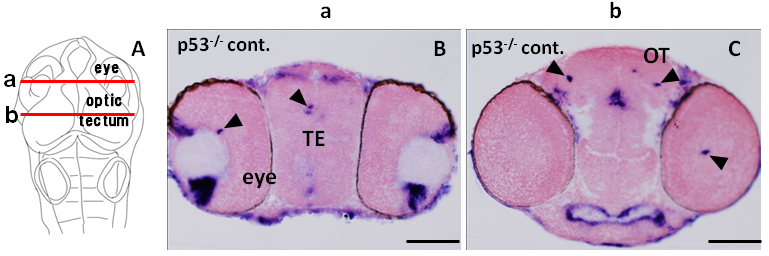

Supplement: S6 Fig — ApoE-expressing cells were demonstrated by WISH in nonirradiated p53-/- embryos. Frontal plastic sections including the eyes and the OT of WISH-stained p53–/–embryos at the ‘a’ and ‘b’ levels of the brain are shown in B and C, respectively. A small number of ApoE-positive cells were present in the retina (arrowheads in A and B), telencephalon (TE; arrowheads in A), and optic tectum (OT; arrowhead in B). Scale bars = 50 μm. (TIF) [file pone.0127325.s006.tif]
